# Tube Flow Shunt: An Innovative and Cost-Effective Approach to the Modified Bleb-Forming Filtration Technique

**DOI:** 10.7759/cureus.82399

**Published:** 2025-04-16

**Authors:** Md Iftekher Iqbal

**Affiliations:** 1 Department of Ophthalmology, Bangladesh Eye Hospital, Dhaka, BGD; 2 Department of Glaucoma, Ispahani Islamia Eye Institute and Hospital, Dhaka, BGD

**Keywords:** ahmed glaucoma valve, ex-press mini-shunt, glaucoma drainage device, glaucoma surgery, modified bleb-forming filtration surgery, refractory glaucoma, tube flow shunt

## Abstract

Aiming to lower intraocular pressure, trabeculectomy remains the gold standard procedure. This report describes a novel, cost-effective modified bleb-forming filtration technique, tFlow (tube flow shunt), utilizing sterile trimmed silicone tubes from the Ahmed Glaucoma Valve (AGV), model FP7, to construct a subconjunctival filtration pathway. Here, clinical outcomes are not reported and will be evaluated in a future study. While initial findings suggest it may provide a viable alternative to other modified bleb-forming filtration techniques involving commercial devices like PreserFlo MicroShunt, EX-PRESS Glaucoma Filtration Device, and XEN Gel Stent, further investigation is necessary. Additional patient inclusion and comparative studies with different bleb-forming filtration techniques are potential areas for future research.

## Introduction

Glaucoma remains the leading cause of irreversible blindness worldwide, with intraocular pressure (IOP) being the only modifiable risk factor. It is estimated that by 2040, over 111.8 million people will be affected by glaucoma globally [1].

For effective control of IOP, trabeculectomy remains the gold standard, while a glaucoma drainage device is the modality of choice for refractory glaucoma. However, they carry significant risks of complications such as hypotony, bleb failure, and fibrosis [2].

The recent development of modified bleb-forming filtration procedures offers a safer alternative with faster recovery times. They have emerged to address the limitations of traditional trabeculectomy while preserving its efficacy in IOP control [3]. Patients with moderate to severe glaucoma and a high risk of progression, despite pharmacological treatment, are the target audience for this design [4]. This method has many benefits, such as a strong effect on lowering IOP with a standard lumen size, a low risk of hypotony, better comfort due to the formation of posterior blebs (which also lowers the risk of inflammation), and less intense postoperative care. On the other hand, cytostatic agents (mitomycin C or 5-fluorouracil) are still required to address wound healing [5,6].

Devices such as PreserFlo MicroShunt (Santen, Osaka, Japan), XEN Gel Stent (Allergan, California, USA), and EX-PRESS Glaucoma Filtration Device (Alcon Laboratories, Texas, USA) have been utilized in surgical practice as a modified bleb-based approach to reduce resistance to aqueous humor outflow, with documented success in lowering IOP and reducing medication burden. However, they remain costly and may present complications such as fibrosis, hypotony, and bleb failure [4,5].

This report introduces tFlow (tube flow shunt), a cost-effective modification of bleb-forming filtration surgery, utilizing a sterile trimmed silicone tube from the Ahmed Glaucoma Valve (AGV) (New World Medical Inc., CA, USA), model FP7. The primary objective is to present the technique and discuss its potential applications, particularly in resource-limited settings, while recognizing that its clinical outcomes are not reported and will be evaluated in future studies.

## Technical report

This surgical approach is currently investigational and being performed under an institutional research protocol with appropriate consent.

Patient selection

Patients included had no perception of light and uncontrolled IOP despite maximum tolerated medical therapy. The primary outcome measures were IOP reduction, medication burden, and tube-related complications.

Surgical technique

**Figure 1:**
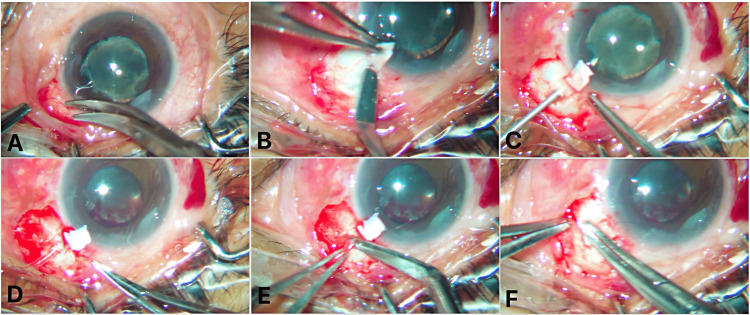
Key surgical steps involved in the tFlow procedure: A) Fornix-based peritomy, B) Partial thickness scleral flap creation, C) 26-gauge needle track through sclera into the AC at the level of ciliary sulcus, D) Securing tube with sclera using 10-0 nylon, E) Tube trimming, and F) Scleral flap edges suturing with 10-0 nylon. tFlow: Tube Flow Shunt; AC: Anterior Chamber.

The tFlow procedure was performed under topical or local anesthesia, with or without a corneal traction suture, using a modified augmented trabeculectomy approach with 0.02% mitomycin C (MMC) to enhance filtration success. The procedure was carried out in the superior, superotemporal, or superonasal quadrant (Figure 1, Video 1).

**Video 1:** tFlow: Tube Flow Shunt

A subconjunctival injection of 0.1 mL of 0.02% MMC was administered and left in place for one minute to modulate wound healing. A fornix-based conjunctival peritomy was created, and hemostasis was achieved with mild cautery. A 4 × 2 mm partial-thickness scleral flap was meticulously dissected to provide coverage for the implanted tube. A 26-gauge needle was used to create a precise track into the ciliary sulcus, ensuring a perpendicular entry to the pupillary plane at a distance of 2 mm from the limbus. A trimmed silicone tube (5mm), collected from a sterile pack of the AGV FP7 model, was inserted through this track into the anterior chamber, facilitating controlled aqueous outflow to the subconjunctival space. The tube was secured to the sclera with 10-0 nylon sutures, ensuring stability and proper positioning to prevent migration. The scleral flap was repositioned and sealed with two 10-0 nylon sutures to regulate outflow resistance. The anterior chamber was reformed using a balanced salt solution, and meticulous evaluation was conducted to confirm the absence of leakage. The conjunctiva was closed in a watertight fashion using an 8-0 polyglactin suture to prevent wound leaks and optimize bleb formation. Finally, an intracameral injection of 0.5 mL of moxifloxacin (0.8 mg/mL) was administered to reduce the risk of postoperative infection.

Postoperative care

Postoperatively, topical prednisolone acetate (1%) was administered hourly for the first postoperative day and then tapered over one month. Additionally, moxifloxacin (0.5%) was prescribed four times daily for one month to prevent infection.

Follow-up

Follow-up evaluations were scheduled based on the surgeon’s discretion, with visits typically occurring on postoperative day one, week one, and at one, three, six, and 12 postoperative months. This tailored follow-up approach allowed for individualized patient monitoring, ensuring proper healing and early detection of any complications. Regular postoperative assessments provided valuable insights into the recovery process and facilitated necessary adjustments to the treatment plan.

## Discussion

Modified bleb-based filtration techniques aim to enhance aqueous outflow by creating a controlled subconjunctival drainage pathway while minimizing complications associated with traditional surgeries. Trabeculectomy, long regarded as the gold standard, involves creating a guarded sclerostomy under a scleral flap but is often associated with risks such as hypotony, bleb leaks, and fibrosis. To address these challenges, the Ex-PRESS glaucoma filtration device was introduced as a refinement, eliminating the need for a surgical sclerostomy and iridectomy by placing a standardized metallic micro-shunt beneath the scleral flap. This approach preserves the outflow mechanism of trabeculectomy while potentially improving predictability and reducing tissue trauma [6]. The PreserFlo MicroShunt, which is composed of poly(styrene-block-isobutylene-block-styrene) to minimize fibrosis, maintains effective aqueous drainage with minimal inflammation [4]. The Ex-PRESS Glaucoma Filtration Device, made of biomedical stainless steel (316L), is stable and MRI-compatible. It has been utilized in surgical practice as a modified bleb-forming approach to reduce resistance to aqueous humor outflow, with documented success in lowering IOP and reducing medication burden [1,5,6]. The XEN Gel Stent, made from porcine gelatin, has also been associated with high needling rates [7].

Compared to these commercial implants, the tFlow procedure builds on the existing EX-PRESS Glaucoma Filtration Device implant concept while addressing cost constraints by utilizing a sterile, trimmed silicone tube of the AGV FP7 model [2,6]. This technique leverages existing surgical skills from trabeculectomy, which can be a practical option for widespread adoption [8]. By physically preserving the patency of the sclerostomy tract with a silicone tube, the tFlow technique may help maintain outflow during early healing, reducing the risk of ostium closure due to fibrosis or iris obstruction. Studies have indicated that MMC plays a crucial role in modulating wound healing in filtration surgeries, and its use in tFlow may indicate its long-term success [4]. However, potential complications such as tube migration, fibrosis, and long-term bleb viability require further study [8]. Before inserting the tube into the anterior chamber, we must trim the tube length accordingly, as the total tube length is 25.4 mm and the tube's inner diameter is 0.305 mm [1]. The trimmed AGV tube used in the tFlow procedure is intended for use in the same surgical case from which it is obtained. No reuse of the tube between different patients has been performed.

To better understand how the tFlow procedure compares with other established bleb-forming glaucoma filtration techniques, a comparative overview is provided in Table 1 [1-3,6,7].

**Table 1:** Comparative table of different bleb-forming filtration techniques AGV: Ahmed Glaucoma Valve; tFlow: Tube Flow Shunt; MMC: Mitomycin C; NA: Not Applicable.

	Trabeculectomy	AGV	Ex-PRESS	PreserFlo	XEN Gel Stent	Deep Sclerectomy	tFlow
Cost	$300–500	$300–500	$400–600	$500–700	$500–700	$300–600	$100-200
Conjunctival peritomy	Yes	Yes	Yes	Yes	Yes	Yes	Yes
Scleral flap	Yes	Yes, or patch graft	Yes	Yes	No	Yes	Yes
Approach	Ab externo	Ab externo	Ab externo	Ab externo	Ab internal (standard); ab externo (optional)	Ab externo	Ab externo
Sclerostomy	Yes	Yes	No	No (uses needle tract)	Yes	No	Yes
MMC use	Yes	Yes	Yes	Yes	Yes	Yes	Yes
Goniolens	No	No	No	No	Yes (if ab interno)	No	No
Lumen size control	Not applicable	Yes	Yes	Yes	Yes	No	Yes (same as AGV)
Needling rate	Moderate	Low	Low	Moderate	Often	Low	Unknown
Visual recovery	4–6 weeks	6–8 weeks	2–4 weeks	2–4 weeks	1–2 weeks	4–6 weeks	Under evaluation
Hypotony risk	High	Moderate	Moderate	Low	Moderate	Low	Unknown
Implant source	NA	Purpose-built	Purpose-built	Purpose-built	Purpose-built	NA	Repurposed
Implant material	NA	Silicone	Stainless steel (316L)	Poly(styrene-block-isobutylene-block-styrene) (SIBS)	Porcine gelatin	NA	Silicone	
Sterility	NA	Factory sterilized	Factory sterilized	Factory sterilized	Factory sterilized	NA	Factory sterilized
Learning curve	Moderate to steep	Moderate	Moderate	Low to moderate	Low	Steep	Moderate

To our knowledge, no previous peer-reviewed reports have described the use of AGV silicone tube remnants in this specific modified bleb-forming approach.

Ultimately, future research should focus on long-term comparative studies between tFlow, PreserFlo, and XEN Gel Stent, analyzing IOP reduction, medication burden, and complication rates over extended follow-up periods [8].

## Conclusions

The tFlow technique is a novel and cost-effective modification of traditional bleb-forming surgery that uses trimmed, sterile AGV tube remnants. While it shares structural similarities with Ex-PRESS implantation, it eliminates the cost and material constraints associated with proprietary devices. This technical report presents the detailed surgical methodology for reproducibility and early adoption in low-resource settings. Clinical effectiveness and safety outcomes remain to be established in future prospective trials. If future studies confirm clinical safety and efficacy, tFlow may serve as a low-cost alternative to proprietary bleb-forming devices in resource-constrained settings.
